# In Vitro Modelling of Osteogenesis Imperfecta with Patient-Derived Induced Mesenchymal Stem Cells

**DOI:** 10.3390/ijms25063417

**Published:** 2024-03-18

**Authors:** Lauria Claeys, Lidiia Zhytnik, Laura Ventura, Lisanne E. Wisse, Elisabeth M. W. Eekhoff, Gerard Pals, Nathalie Bravenboer, Vivi M. Heine, Dimitra Micha

**Affiliations:** 1 Department of Human Genetics, Amsterdam UMC Location Vrije Universiteit Amsterdam, 1081 HV Amsterdam, The Netherlands; l.claeys@amsterdamumc.nl (L.C.); l.zhytnik@amsterdamumc.nl (L.Z.); l.ventura@amsterdamumc.nl (L.V.); l.wisse@amsterdamumc.nl (L.E.W.); g.pals@amsterdamumc.nl (G.P.); 2 Rare Bone Disease Center Amsterdam, 1081 HV Amsterdam, The Netherlands; emw.eekhoff@amsterdamumc.nl; 3 Amsterdam Movement Sciences, 1081 HV Amsterdam, The Netherlands; n.bravenboer@amsterdamumc.nl; 4 Amsterdam Reproduction and Development, 1081 HV Amsterdam, The Netherlands; 5 Department of Traumatology and Orthopaedics, The University of Tartu, 50410 Tartu, Estonia; 6 Department of Endocrinology and Metabolism, Amsterdam UMC Location Vrije Universiteit, 1081 HV Amsterdam, The Netherlands; 7 Department of Clinical Chemistry, Amsterdam UMC Location Vrije Universiteit Amsterdam, 1081 HV Amsterdam, The Netherlands; 8 Department of Child and Adolescent Psychiatry, Amsterdam UMC Location Vrije Universiteit Amsterdam, Amsterdam Neuroscience, 1081 HV Amsterdam, The Netherlands; vm.heine@amsterdamumc.nl; 9 Department of Complex Trait Genetics, Center for Neurogenomics and Cognitive Research, Vrije Universiteit Amsterdam, Amsterdam Neuroscience, 1081 HV Amsterdam, The Netherlands

**Keywords:** induced mesenchymal stem cells, cell model, osteoblast, bone, skeletal dysplasia, Osteogenesis Imperfecta

## Abstract

(1) Mesenchymal stem cells (MSCs) are a valuable cell model to study the bone pathology of Osteogenesis Imperfecta (OI), a rare genetic collagen-related disorder characterized by bone fragility and skeletal dysplasia. We aimed to generate a novel OI induced mesenchymal stem cell (iMSC) model from induced pluripotent stem cells (iPSCs) derived from human dermal fibroblasts. For the first time, OI iMSCs generation was based on an intermediate neural crest cell (iNCC) stage. (2) Skin fibroblasts from healthy individuals and OI patients were reprogrammed into iPSCs and subsequently differentiated into iMSCs via iNCCs. (3) Successful generation of iPSCs from acquired fibroblasts was confirmed with changes in cell morphology, expression of iPSC markers *SOX2*, *NANOG*, and *OCT4* and three germ-layer tests. Following differentiation into iNCCs, cells presented increased iNCC markers including *P75NTR*, *TFAP2A*, and *HNK-1* and decreased iPSC markers, shown to reach the iNCC stage. Induction into iMSCs was confirmed by the presence of *CD73*, *CD105*, and *CD90* markers, low expression of the hematopoietic, and reduced expression of the iNCC markers. iMSCs were trilineage differentiation-competent, confirmed using molecular analyses and staining for cell-type-specific osteoblast, adipocyte, and chondrocyte markers. (4) In the current study, we have developed a multipotent in vitro iMSC model of OI patients and healthy controls able to differentiate into osteoblast-like cells.

## 1. Introduction

Osteogenesis Imperfecta (OI) is a rare genetically heterogeneous connective tissue disorder characterized mainly by fragile bones and skeletal dysplasia. Up to 85% of patients have a pathogenic variant in the *COL1A1* or *COL1A2* gene which code for two collagen chain α1 (I) and one collagen chain α2 (I), respectively, forming the triple helix of the collagen type I protein. Hundreds of different pathogenic variants have been identified in the collagen type I genes, leading to a wide range of OI phenotypes. Collagen type I pathogenic variants can be divided into two categories based on their molecular effect, namely haploinsufficiency (HI), originating from whole-gene deletions, nonsense, and frameshift mutations, and the dominant negative (DN) defect, arising from missense mutations [1]. Patients with HI of collagen type I produce a reduced amount of protein (i.e., quantitative defect), while patients with DN pathogenic variants produce structurally abnormal collagen type I (i.e., qualitative defect). HI results mostly in milder OI phenotypes (OI type 1), while the DN defects most often lead to more severe phenotypes (OI type 2–4). Considering that collagen type I is produced by osteoblasts, they are the responsible cell type for OI presentation in bone. Their crucial anabolic role in the maintenance of bone mass, necessitates their study for the in-depth understanding of OI pathophysiology [2].

Osteoblasts can be obtained from bone tissue using invasive procedures like bone biopsies and orthopedic surgeries, which entail distress and risks to the patient. Moreover, osteoblasts can be directly differentiated from mesenchymal stem cells (MSCs) found in many different tissues (e.g., bone marrow, adipose tissue, umbilical cord blood, amniotic fluid, placenta, dental pulp, tendons, synovial membrane, and skeletal muscle) [3]. However, primary MSCs have a number of limitations restricting their use in the research of rare bone disorders. These include their limited cell proliferative capacity and differentiation potential during long-term culture [4,5] as well as the scarce availability of donors, isolation and expansion challenges. In addition, there is significant donor-specific variation, which affects the proliferation and differentiation potential of the cells [4,6,7]. In order to circumvent the aforementioned limitations, efforts have been made to generate bone cell models from the less invasively obtained skin fibroblasts, a collagen-producing cell type, which can mimic the disease mechanism [8]. However, fibroblasts fail to differentiate into distinct mesenchymal lineages. As an alternative, induced mesenchymal stem cells (iMSCs) were employed to overcome these limitations [9,10]. The iMSCs have infinite growth and differentiation properties, adapting these cells to maintenance in long-term culturing and gene editing [4]. Furthermore, iMSCs can be used not only for the study of the disease’s pathophysiology but also for drug screening and cell therapy purposes [4,11,12,13,14]. At least six clinical trials investigating the safety, tolerability, and efficacy of iMSC exist to date [9]. There is still no consensus about the assessment of the clinical quality and safety of this new cell type; thus, general MSC criteria are applied to iMSCs as well.

The generation of iMSCs from fibroblasts can be performed via different pathways. By transfecting the “Yamanaka factors” Klf4, Sox2, Oct3/4, and c-Myc, fibroblasts are reprogrammed into induced pluripotent stem cells (iPSCs) [15]. The iPSCs can then be directed towards iMSCs based on different protocols [4]. One approach is to expose the iPSCs, seeded either as a monolayer or grown into embryoid bodies, to variations of MSC culture medium, such as Dulbecco’s Modified Eagle Medium (DMEM) or α-Minimum Essential Medium (α-MEM) with 10–15% fetal bovine serum (FBS) [12,13,15,16,17,18]. However, iMSCs developed in such a direct way showed a diminished differentiation potential [4]. On the contrary, the imitation of the natural embryonic development of MSCs leads to the generation of iMSCs with more predictable properties and functions [4]. Although the developmental origin of MSCs is still not completely understood, based on the latest studies, it can be hypothesized that MSCs are derived from the lateral plate mesoderm, from which most adipose and skeletal tissues of the body originate [3]. Nevertheless, in the embryonic trunk stage, MSC-like cells are formed from the neuroepithelium through a neural crest intermediate stage [3,19]. Neural crest cells (NCCs) originate at the interface between the neural tube and the epidermis and migrate extensively to form a plethora of different cells besides the mesenchymal precursor cells such as neurons, glia, melanocytes, and endocrine cells [20,21,22]. The NCCs forming the mesenchymal precursor cells most likely derive from the cranial neural crest [21]. This has stimulated the development of protocols for the differentiation of iPSCs into iMSCs through NCC lineage [4,23,24]. In this process, the activation of the canonical Wnt signaling pathway and concomitant suppression of the Activin A/Nodal and Bone Morphogenetic Protein (BMP) signaling, also called dual SMAD inhibition, is critical for driving the pluripotent stem cells into the induced neural crest cells (iNCC) stage [20,23,25]. Wnt signaling can be activated by the addition of a small molecule inhibitor of glycogen synthase kinase 3 (GSK3) such as (2′Z,3′E)-6-bromoindirubin-3′-oxime (BIO) or CHIR99021 [23]. BMP signaling is inhibited by Noggin or Dorsomorphin by binding to the activin-receptor like kinase (ALK)1/2/3/6. Finally, the Activin A/Nodal/TGFβ signaling can be suppressed by SB431542 via blocking the phosphorylation of the ALK4/5/7 receptors [23,25,26,27]. The subsequent differentiation into iMSCs is achieved by administering MSC medium, namely αMEM with FBS and fibroblast growth factor 2 (FGF-2) [4,28].

In this study, we aimed to develop a multipotent iMSC model based on patient-derived iPSCs reprogrammed from dermal fibroblasts with DN and HI *COL1A1* pathogenic variants. iMSCs from three healthy control and three OI patient cell lines were generated for the first time via the iPSC-iNCC intermediate stage. The developed iMSCs were subsequently differentiated into functional osteoblast-like cells, as confirmed by gene expression of cell-type-specific markers and mineralization assay.

## 2. Results

### 2.1. Morphological Modifications of Induced Pluripotent Stem Cells, Induced Neural Crest Cells, and Induced Mesenchymal Stem Cells

**Figure 1:**
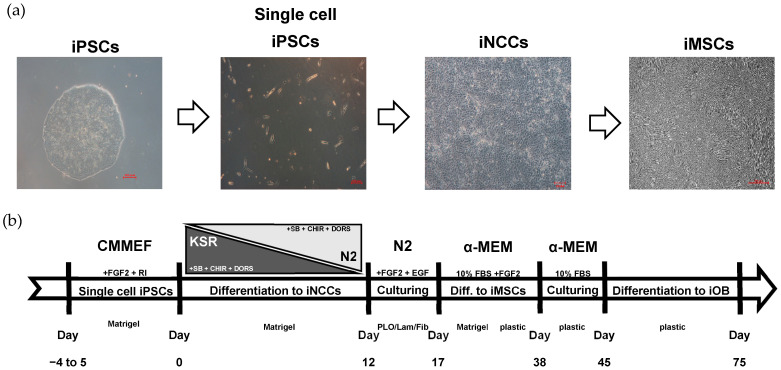
Differentiation of induced pluripotent stem cells (iPSCs) into induced mesenchymal stem cells (iMSCs) via induced neural crest cells (iNCCs) stage. (**a**) Micrographs showing the cell morphology of the subsequent differentiation stages from iPSCs (scale bar 100 µm) into single cell iPSCs (scale bar 100 µm) iNCCs (scale bar 100 µm) and iMSCs (scale bar 200 µm). (**b**) The visual representation of a protocol of the differentiation from iPSCs to iNCCs and iMSCs.

Fibroblasts of three healthy donor control cell lines (C1, C2 and C3), one HI OI patient (P1), and two DN OI patients (P2 and P3) were differentiated into iPSCs (Table S1). iPSCs present typical compact colonies with distinct borders. After neural induction of the iPSCs’ single cell culture, cells gradually proliferated and changed morphologically into larger slightly triangular cells with some protrusions. During mesenchymal induction, cells elongated into fibroblast-like spindle-shaped cells (Figure 1a). No difference in morphology was observed between the patient (P1, P2, and P3) and control (C1, C2, and C3) cell lines.

### 2.2. Characterization and Comparison of Induced Pluripotent Stem Cells to Primary Embryonic Stem Cells

**Figure 2:**
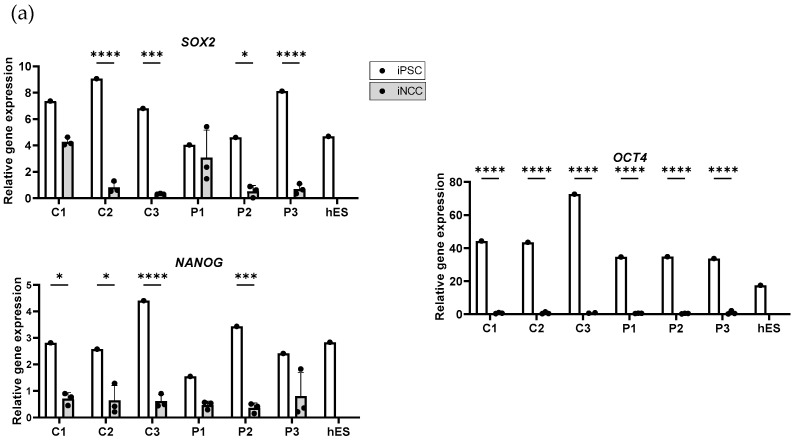

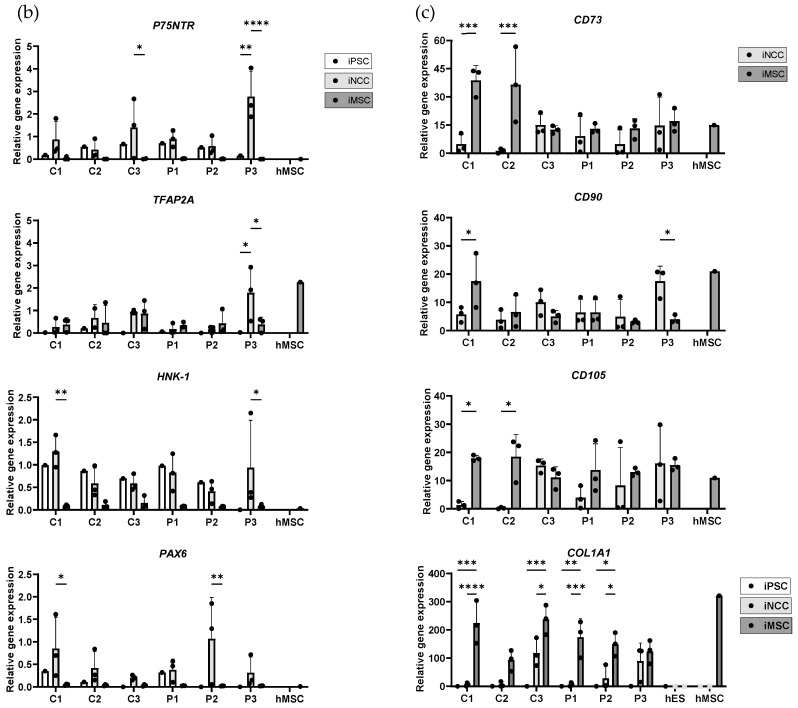
Relative mRNA expression of cell-type-specific marker expression in induced pluripotent stem cells (iPSCs), induced neural crest cells (iNCCs), and induced mesenchymal stem cells (iMSCs). (**a**) Corresponding iPSCs and iNCCs of healthy controls (C1, C2, C3), OI patients (P1, P2, and P3), and a human embryonic stem cell line (hES) were compared for mRNA expression of pluripotent stem cell markers *SOX2*, *NANOG*, and *OCT4*. (**b**) Relative mRNA expression levels of corresponding iPSCs, iNCCs, and iMSCs (C1, C2, C3, P1, P2, and P3) and primary bone-marrow MSCs (hMSCs) were compared for expression of iNCC cell markers *P75NTR*, *HNK-1*, and *TFAP2A* and neuro-ectoderm marker *PAX6*. (**c**) mRNA expression levels of corresponding iNCCs and iMSCs and hMSCs were compared for iMSC markers *CD73*, *CD105*, and *CD90*. Relative mRNA expression levels of the *COL1A1* gene were also shown for iPSCs, iNCCs and iMSCs, hMSC and hES. Gene expression is the normalized relative expression against *TBP*. Standard deviation (SD) is shown per iNCC and iMSC group (n = 3), no SD is present for iPSCs, hES and hMSC (n = 1). Statistical significance is indicated on the graphs (*p*-values ≤ 0.05 (*), ≤0.01 (**), ≤0.001 (***), ≤0.0001 (****)).

RT-qPCR analysis of the pluripotency genes SRY-Box Transcription Factor 2 (*SOX2*), Homeobox Transcription Factor Nanog (*NANOG*), and Octamer-Binding transcription factor 4 (*OCT4*) revealed expression levels in the OI patient and control iPSCs lines comparable to expression of these markers in human embryonic stem cells (hES) (Figure 2a). Characterization of the iPSCs using immunocytochemistry, stainings for OCT4, T cell receptor alpha locus 60 (TRA-1-60), T cell receptor alpha locus 81 (TRA-1-81), and the 3-germ layer test (data not shown) demonstrated the successful reprogramming of the fibroblasts into iPSCs of all six cell lines. A routine Global Screening Array (GSA) did not show major allelic changes in the generated iPSC clones, whereas the presence of pathogenic OI variants (Table S1) in OI iPSCs was confirmed with Sanger sequencing (data not shown).

### 2.3. Characterization of Induced Neural Crest Cells

Overall downregulation of *SOX2*, *NANOG*, and *OCT4* was observed in the iNCCs compared to the iPSCs (Figure 2a) supporting the neural crest induction of the six former iPSC cell lines. The NCC expression profile was confirmed with the upregulation of Low Affinity Neurotrophin Receptor P75NTR (*P75NTR*), Transcription Factor AP-2 Alpha (*TFAP2A*), and paired box 6 (*PAX6*) in iNCCs compared to the iPSCs (Figure 2b). In contrast to our expectations, human natural killer-1 (*HNK-1*) expression did not display any difference in iNCCs compared to iPSCs (Figure 2b). High heterogeneity in the expression of iPSC and NCC markers by iNCC was observed between different cell lines and also between induction rounds.

**Figure 3:**
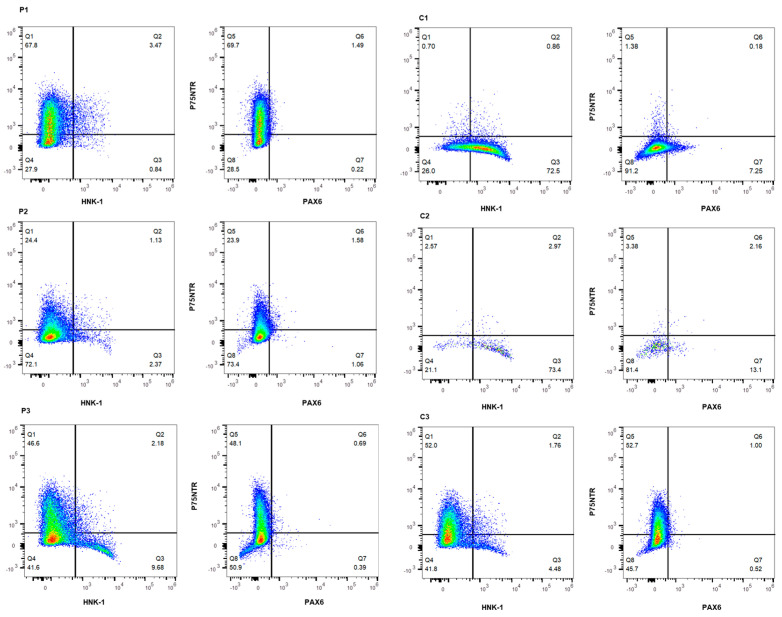
Induced neural crest cells (iNCCs) marker expression analysis using flow cytometry. Flow cytometry profiles of iNCC markers P75NTR (*y*-axis) and HNK-1 (*x*-axis) and neuro-ectoderm marker PAX6 (*x*-axis) in iNCC cells (healthy controls (C1, C2, C3), OI patients (P1, P2, and P3)). Q1 indicates the P75NTR+ cells and Q2 indicates the P75NTR+/HNK-1+ and P75NTR+/PAX6+ cells, respectively; Q3 presents the HNK-1+ and PAX6+ cells, respectively; and Q4 presents the P75NTR-/HNK-1− and P75NTR−/PAX6− cells, respectively.

Flow cytometry (FACS) showed a substantial P75NTR+ cell population in all but two out of six cell lines (C1 and C2) in which, in comparison to the other four cell lines, a significant part of the cell population presented HNK-1+ (72.5% and 73.4%, respectively) (Figure 3, Table 1). It has to be noted, that the total number of cells was modest for C2 (Figure 3). Almost no P75NTR+/HNK-1+ cells were observed in any of the cell lines (Figure 3, Table 1). Additionally, scarcely any PAX6 positive cells were present (Figure 3, Table 1). In combination with the gene expression data, flow cytometry findings confirmed the iNCC phenotype as indicated by the considerable presence of P75NTR+, HNK-1+, and scarce PAX6+ cell populations.

### 2.4. Characterization and Comparison of Induced Mesenchymal Stem Cells to Primary Human Mesenchymal Stem Cells and Hematopoietic Stem Cell

After the mesenchymal induction of iNCCs into iMSCs *P75NTR*, *HNK-1* and *PAX6* relative gene expression dropped, whereas *TFAP2A* expression remained to be overall consistent (Figure 2b). The relative expression of MSC-specific markers *CD73*, *CD105*, and *CD90* in generated iMSCs was equivalent to primary human bone marrow mesenchymal stem cells (hMSC) (Figure 2c). Furthermore, all three markers were already detectable at the iNCC stage, however, with a lower expression degree. The human hematopoietic stem cell (hHSC) expression profile (*CD45*, *CD14*, *CD34*, and *CD79α*) was significantly lower in the healthy control and patient iMSCs, as well as in hMSCs in comparison to hHSCs, with the exception of *CD34* expression in C3 and the hMSCs (Figure S1). As expected, all iMSC cell lines and hMSC intensively expressed *COL1A1*, whereas it was moderately expressed in iNCC cell lines and almost undetectable in iPSCs and hES (Figure 2c). A trend of lower expression of *COL1A1*/*COL1A2* ratio in HI OI patient cell lines compared to control iMSCs was observed (data not shown). Similarly to iNCCs, iMSCs showed greater variability compared to iPSC cell lines.

### 2.5. Osteogenic Induction and Mineralization Assay of OI and Healthy Control Induced Mesenchymal Stem Cells

**Figure 4:**
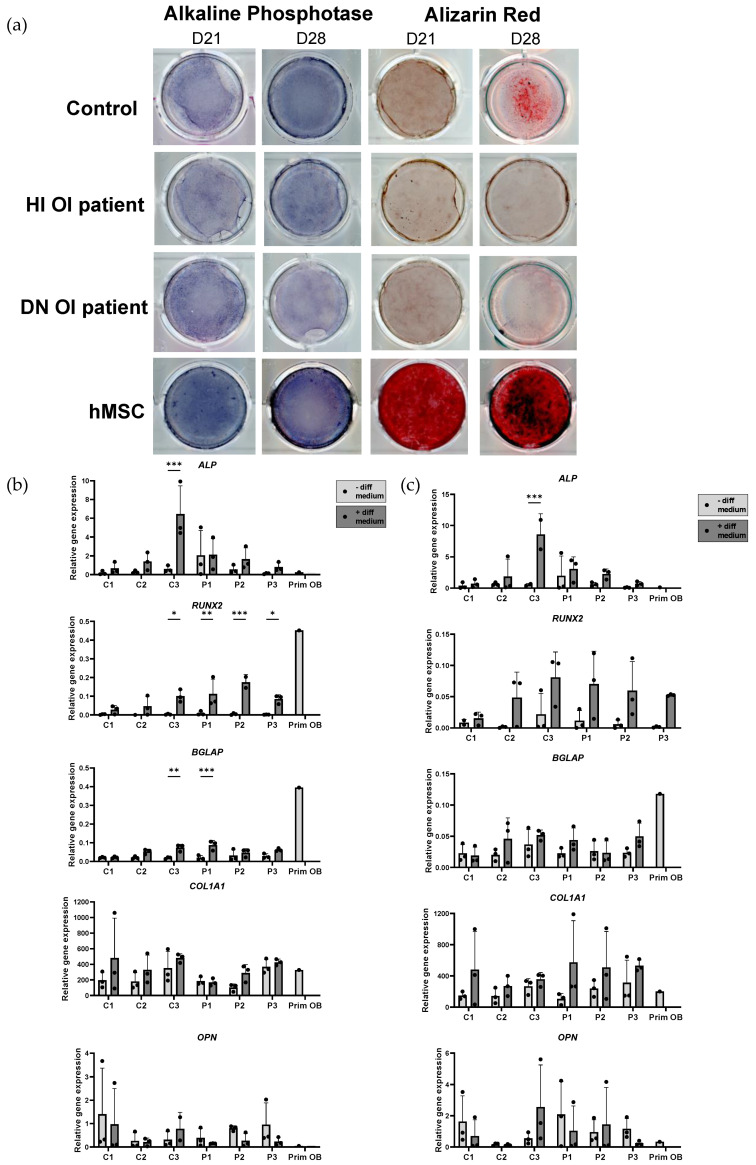
Characterization of induced osteoblasts. (**a**) Osteogenic induction of induced mesenchymal stem cells (iMSC) cell lines was analyzed with Alizarin Red (AR) staining for calcium deposition and Alkaline Phosphatase (ALP) staining to measure osteoblast activity on day 21 and day 28. Representative images for control, haploinsufficient (HI) patient and dominant negative (DN) patient are shown. Relative mRNA expressions of osteoblast (*ALP*, *RUNX2*, *BGLAP*, *OPN*, *COL1A1)* and mesenchymal stem cell (MSC) markers (*CD73*, *CD105*, and *CD90*) were compared in non-differentiated and differentiated iMSCs (healthy controls (C1, C2, C3), OI patients (P1, P2, and P3)), and primary osteoblasts (Prim OB) on (**b**) day 21 and (**c**) day 28 of the differentiation). Relative gene expression was normalized against *TBP*. Standard deviation (SD) is presented per cell line (n = 3); no SD is present for the primary osteoblasts (n = 1). Statistical significance is indicated on the graphs (*p*-values ≤ 0.05 (*), ≤0.01 (**), ≤0.001 (***)).

In response to osteogenic induction, iMSC cell lines showed an increase in alkaline phosphatase (ALP) activity compared to non-induced cells on both day 21 and 28 (Figure 4a, Table S2). Alizarin red staining confirmed the presence of mineralization of both patient and control iMSCs on day 21 and 28 to a certain extent (Figure 4a, Table S2). Reduced mineralization of patient iMSCs compared to control iMSC cell lines was seen on day 28. On day 21 and 28, the hMSCs showed higher mineral deposition in vitro, compared to iMSC cell lines (Figure 4a).

Gene expression analysis revealed the presence of early and mature osteoblast markers *ALP*, *RUNX* family of transcription factor 2 (*RUNX2*), *COL1A1*, and bone gamma-carboxyglutamate protein (*BGLAP*) on day 21 and 28 after the osteogenic induction of both control and patient cell lines (Figure 4b,c). The expression of osteopontin (*OPN*) did not change, or reduce as a consequence of osteogenic induction (Figure 4b,c). No difference in the expression of the MSC markers *CD73* and *CD90* between iMSCs with or without osteogenic induction at day 21 and 28 was observed (Figure S2A,B). Generally, OI iMSCs showed higher expression of *CD105* on day 21 compared to the control iMSCs (Figure S2A). At the same timepoint, a significant increase in the relative gene expression of *CD105* caused by osteogenic induction in patient cell lines (P2 and P3) was detected.

### 2.6. Adipogenic and Chondrogenic Induction of OI and Healthy Control Induced Mesenchymal Stem Cells

**Figure 5:**
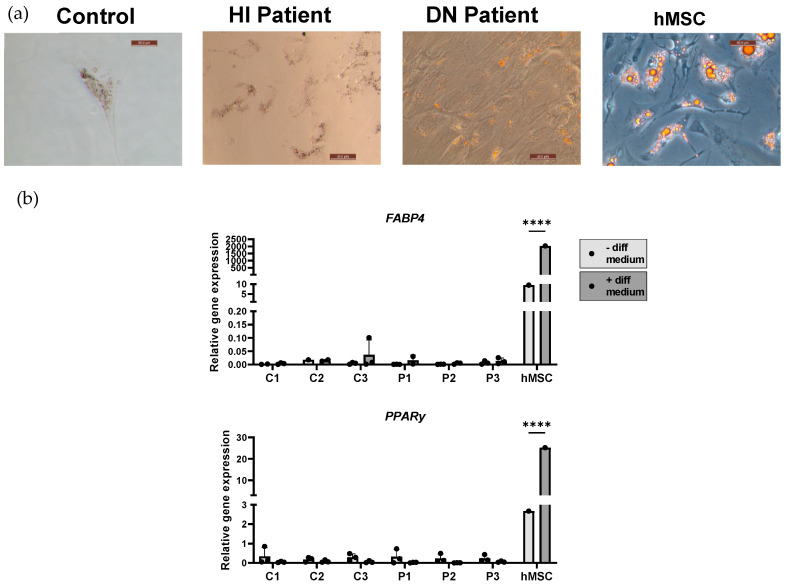
Characterization of induced adipocytes. (**a**) Adipogenic differentiation of induced mesenchymal stem cells (iMSC) cell lines was analyzed with Sudan III staining, where orange indicates stained lipid vesicles (scale bar size 50 µm). Representative images of control, haploinsufficient (HI) patient, dominant negative (DN) patient, and primary bone marrow mesenchymal stem cells (hMSC) are shown. (**b**) Relative mRNA expression of adipocyte markers (*FABP4* and *PPARy*) was compared in non-differentiated and differentiated iMSCs (healthy controls (C1, C2, C3), OI patients (P1, P2, and P3)), and hMSC. Relative gene expression was normalized against *TBP*. Standard deviation (SD) is shown per cell line (n = 3); no SD is present for hMSC (n = 1). Statistical significance is indicated on the graphs (*p*-values ≤ 0.0001 (****)).

Adipocyte differentiation was verified with the formation of lipid vesicles stained with Sudan III, following iMSCs culturing with adipocyte differentiation medium (Figure 5a). Following differentiation, a clear difference in morphology between the two conditions was noted. Compared to hMSCs, which achieved a mature adipocyte stage, lipid vesicles formed in iMSCs were mostly of small size, indicating pre-adipocyte stage of the iMSCs (Figure 5a, Table S3). This is corroborated by the low expression level of Fatty Acid-Binding Protein 4 (*FABP4*) and Peroxisome Proliferator-Activated Receptor-Gamma (*PPARy*) compared to hMSCs (Figure 5b). Although, on the gene expression level, no differences were present between patient and control cell lines, higher number of lipid vesicles were present in OI cell lines compared to healthy controls (Table S3).

**Figure 6:**
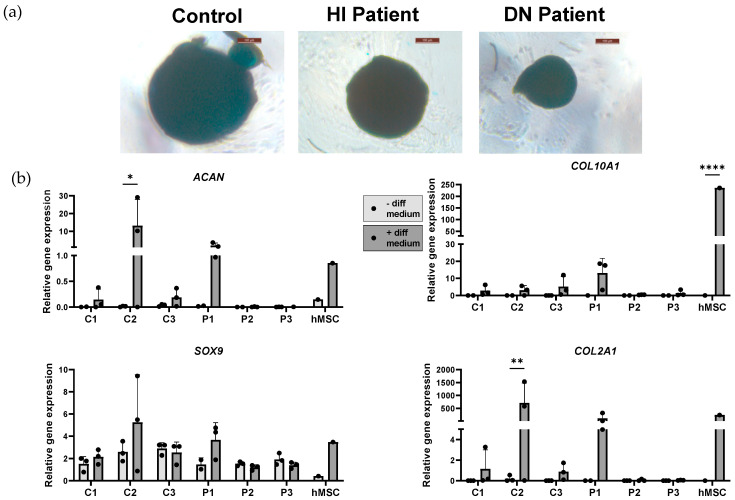
Validation of induced chondrocyte phenotype. (**a**) Chondrogenic differentiation of induced mesenchymal stem cells (iMSC) lines was analyzed with Alcian blue; blue staining indicates sulfated proteoglycans deposits (scale bar size 100 µm). Representative images for control, haploinsufficient (HI) OI patient, dominant negative (DN) OI patient, and primary bone marrow mesenchymal stem cells (hMSC) are shown. (**b**) Relative mRNA expression of chondrocyte markers (*ACAN*, *SOX9*, *COL10A1*, and *COL2A1*) was compared in non-differentiated and differentiated iMSCs (healthy controls (C1, C2, C3), OI patients (P1, P2 and P3)), and hMSC. Relative gene expression was normalized against *TBP*. Standard deviation (SD) is shown per cell line (n = 3); no SD is present for hMSC (n = 1). Statistical significance is indicated on the graphs (*p*-values ≤ 0.05 (*), ≤0.01 (**), ≤0.0001 (****)).

Chondrocyte differentiation was validated using Alcian blue positive micromasses. A blue staining of micromasses after chondrogenic induction was spotted (Figure 6a, Table S4). After stimulating the iMSCs with chondrocyte differentiation medium, a trend of chondrocyte marker (*ACAN*, *SOX9*, *COL2A1*, and *COL10A1*) upregulation was detected. A general tendency of reduced expression of chondrogenic markers in DN OI patients, but not in HI OI and control cell lines, can be appreciated (Figure 6b).

## 3. Discussion

In the present study, we have established an in vitro induced cell model for OI via the reprogramming of fibroblasts into iPSCs with the subsequent differentiation into iNCCs, and eventually iMSCs. To our knowledge, we demonstrate for the first time the use of an iNCC intermediate stage for the generation of OI iMSCs. The model was validated using gene expression analysis and trilineage (osteoblast-, adipocyte-, chondrocyte-like cells) differentiation competence. This model can be utilized for the study of osteoblast differentiation and mineralization, regulation of collagen type I synthesis, as well as testing potential therapeutic interventions in an OI-specific molecular background. World biobanks of OI osteoblast/MSC cell models are scarce, whereas fibroblast cell models do not fully represent the pathophysiological state of OI in mineralized tissue. Thus, it is crucially important for the development of pharmacological and advanced therapies of OI and other rare bone disorders to explore alternative cellular differentiation models like iMSCs, which are an invaluable source of human patient material.

Following the differentiation of iPSCs into iNCCs, an increase in the expression of the iNCC gene *(P75NTR*, *TFAP2A)* and protein (P75NTR, HNK-1) markers emerged, with modest expression of *PAX6* for both mRNA and at protein level. During embryonic development, neural crest stem cell populations are diverse in the expression of NCC markers depending on their phase of development. It has been demonstrated that migratory NCCs are high in P75NTR and TFAP2A expression, while only a few of these cells express HNK-1 [29]. This may indicate that iPSCs may be potentially prone to producing a heterogeneous iNCC population in response to culture for iNCC derivation. This can be potentially explained by the gene expression heterogeneity of iPSCs inside a colony, influence of culture confluency, and difference in exposure to growth factors during differentiation. Future efforts will focus on achieving a pure iNCC population, such as cranial NCCs needed for mesenchymal lineage differentiation, as this might result in a lower variability on the iMSC stage [30].

The MSC markers *CD105*, *CD73*, and *CD90*, included in the MSC-defining criteria of the ISCT, were expressed in the differentiated iMSCs. Additionally, a lack of or reduced expression of the hematopoietic stem cell markers *CD45*, *CD34*, *CD14*, and *CD79α* was also successfully demonstrated in all cell lines. Interestingly, the MSC markers *CD73*, *CD105*, and *CD90* were also present in iNCCs. This is in line with the study of Menendez et al. demonstrating, using FACS analysis, the presence of CD105 and CD90 markers in iNCCs before differentiation into iMSCs; Curchoe et al. also indicated that around 13% of early migratory neural crest stem cells expressed CD73 [23,31]. Although an overall decrease in iNCC markers was noted in our iMSCs lines, the relative gene expression of *TFAP2A* was still present at some level. Fan et al. demonstrated that TFAP2A enhances the osteogenic differentiation potential of MSCs, and knock-out *TFAP2A* mouse models have shown craniofacial defects; hence, it can be speculated that a certain level of TFAP2A may contribute to the osteogenic potential of the iMSCs [32,33,34].

This study demonstrated variability between cell lines at the iNCC and iMSC stage. The largest difference in iNCC marker expression was noticed in the FACS analysis between control cell lines C1, C2, and the rest of iNCC cell lines. C1 and C2 were mostly HNK-1-positive in comparison to the other cell lines, which were instead detected to be mainly P75NTR-positive. Interestingly, these two cell lines at the iMSC stage also demonstrated higher relative expression levels of *CD73* and *CD105* compared to the other cell lines, suggesting that the iNCC stage properties can affect the differentiation into iMSCs. This finding did not translate further to the trilineage differentiations of the iMSCs. A possible reason for the distinct properties of these two control cell lines (C1 and C2) could be the different reprogramming methods used for iPSC generation, namely lentivirus (C1 and C2) and Sendai virus (C3, P1, P2, and P3). A study of Nishino et al. presented evidence of epigenetic differences between reprogramming vectors for iPSCs. The use of the Sendai virus was able to maintain the epigenetic profile of the iPSCs closer to those of embryonic stem cells compared to retrovirus-reprogrammed iPSCs (lentivirus) [35]. As such, epigenetic profiling can be one of the key elements affected following differentiation of direction and the capacity of the cell line. In order to improve the quality of induced stem cell cultures, the effect of the epigenetics should be further investigated and taken into account. However, the difference in reprogramming methods did not translate into variability in our trilineage differentiations, and did not appear to effect other iMSC characteristics.

The differentiation properties of iMSCs were investigated using trilineage differentiation into osteoblasts, adipocytes, and chondrocytes. We demonstrated that the iMSCs differentiated into early osteoblast-like cells based on the increased expression of the early osteoblast markers *RUNX2* and *ALP* and a notable trend of increased mRNA expression of the late osteoblast markers *BGLAP* and *COL1A1* after osteogenic induction at day 21 and 28. This was also confirmed with the overall increased osteoblast activity demonstrated with ALP staining (Figure 4a). All cell lines stained positive for Alizarin red, and the presence of mineralized nodules was noted. However, the mineralization of the iMSCs was reduced compared to the primary bone marrow-derived hMSCs. Variability was noted between the cell lines after osteogenic induction. This could be a result of the variability at the iMSC stage between the different cell lines and the subsequent level of osteogenic differentiation which affected mineralization. The osteogenic differentiation into osteoblast-like cells was performed with human platelet lysate which has been shown to promote the MSC expansion rate without compromising their differentiation capacity [36,37]. Nonetheless, studies about the use of human sera are rare; hence, it is still not completely known which factors are present and how they interact with MSC biology [36].

The iMSCs displayed decreased the adipogenic potential compared to bone marrow MSCs. Although iMSCs morphologically resembled adipocytes, the vesicles were smaller and the gene expression of adipocyte-specific markers was lower compared to hMSCs. It could be hypothesized that they do not differentiate any further than an early-adipocyte stage. In contrast, the iMSCs were capable of differentiating to chondrocytes, as demonstrated with the increased gene expression of chondrocyte markers and the positive Alcian blue staining. Our data indicate a preference of the iMSCs to differentiate into osteoblast-like cells and chondrocytes. The reduced trilineage potential and variability has been demonstrated before in previous iMSC studies and may be explained by the parental origin of the iPSCs and iMSC generation methodology [38,39,40]. The creation and comparison of iMSCs from fibroblasts from the gingiva, lung, and periodontal ligament showed differences at the iMSC stage and trilineage differentiation potential [16]. Regarding the impact of iMSC generation methodology, Glaeser et al. compared iMSCs of two different methodologies, namely iMSCs generated directly from iPSCs and iMSCs generated via an iNCC stage, to compare their potential to promote bone formation in cranial defects in vivo. They demonstrated that the iNCC-iMSC coated allografts promoted a more efficiently repair of cranial bone defect than the iMSC generated directly from iPSCs [13,41]. Finally, variability in the potential for trilineage differentiation is enriched by the heterogeneity of the iMSC population. Inter-individual disparities between our developed iMSCs after osteogenic, adipogenic and chondrogenic induction may also offer the opportunity to explore and understand personalized differences via this human in vitro model.

Primary MSCs have the advantage of being an unmodified patient-specific cell model to investigate patient-specific pathologies and testing of therapies. However, they certainly can also present drawbacks such as heterogeneity which can be instigated using inherited tissue memory and the effect of the microenvironment and the cell population in different differentiation stages [9,10]. This can be exacerbated by differences in handlings and culture conditions by ensuing a potential bias to certain cell types [10]. Additionally, the collection of MSCs is invasive and low-yield, depending from which tissue they are harvested, and the access to patients is limited. Also, bone marrow MSCs lose their proliferation and differentiation potential in vitro much faster than iMSCs, and the quality of the cells is dependent on the age of the donor and can be variable [9,42]. As such, the iMSCs are a captivating alternative source. It has been demonstrated that the reprogramming to iPSCs rejuvenates the iMSCs and results in reduced heterogeneity compared to primary MSCs [9]. The iPSCs can be generated from many different easily acquired cell types, such as skin fibroblasts, and can be used to upscale iMSCs [4]. As aforementioned, although these advantages nominate iMSCs as a good alternative to primary MSCs, several obstacles still need to be addressed, such as the iMSC heterogeneity based on the parental tissue and generation method. Still, as OI is a very genetically and phenotypically heterogeneous disease, the creation of OI patient-specific iMSCs is crucial to investigate the patient-specific underlying pathology and to test precision therapies.

In the current study, we have successfully created iMSCs and subsequently osteoblast-like cells from a healthy control and OI patient fibroblasts. iMSCs show a difference in the adipogenic potential between the patients and control cell lines. On average, the patient cell lines presented more and larger lipid vesicles compared to the control cell lines. Our results are in line with findings in the Brtl mouse, a model for moderate to severe DN OI, in which the skewing of MSCs to adipocytes instead of osteoblasts was observed [43]. This also correlates with conclusions of Kareto et al., who showed an increase in the expression of adipocyte function genes and thus hypothesized increased adipogenic differentiation of OI bone marrow MSCs at the cost of the osteogenic differentiation pathway [44]. A trend of reduced chondrogenic expression in DN OI chondrocyte-like cells induced from iMSCs aligns with deficient growth plate chondrocytes in OIM mice with DN OI, but not HI OI Mov13 mice [45,46].

OI iPSC-iMSC generation has been previously performed in limited studies, all of which made use of direct iMSC induction from embryoid bodies [47,48,49,50]. Although differences in the trilinear differentiation potential of control and OI iMSC cannot be appreciated to the fullest extent in these studies due to lack of staining and/or gene expression data, our study shares in common the identification of lower osteogenic differentiation capacity in the developed OI iPSC-iMSCs.

Further investigation is needed to evaluate iMSC suitability for OI research, including patient-specific differences and the stability of iMSC and subsequent osteoblast-like cell properties in long-term cultures. Additionally, the iMSC characterization can benefit from assessment of MSC markers with FACS analysis, to improve the consistency of the results and allow better evaluation of the OI pathology with this cell differentiation model. Furthermore, MSCs are also known for their paracrine properties, which have been demonstrated to alter the tissue microenvironment to aid in tissue repair [51,52]. As such, it would be interesting to measure paracrine factors in the conditioned media to ensure that the iMSCs have the same properties as MSCs. Finally, it is necessary to characterize and investigate the differences in bone regeneration potential between the control iMSCs and the different patient-derived iMSCs in 3D and in vivo environments.

## 4. Materials and Methods

### 4.1. Cells

Primary dermal fibroblasts were obtained from skin biopsies of patients. Permission was granted beforehand by the local medical ethics committee (Amsterdam UMC, VUmc) and all experiments were performed in accordance with local guidelines and regulations. Informed consent was obtained from all participants and/or their legal guardians. Primary human dermal fibroblasts were cultured in Ham’s F-10 Nutrient Mix medium (Gibco, Grand Island, NY, USA, 31550-031) supplemented with heat-inactivated 10% FBS (Gibco, Grand Island, NY, USA, 10270-106) and 1% penicillin/streptomycin (PEN/STREP, Gibco, Grand Island, NY, USA, 15140-122). All cell lines used in this study tested negative for mycoplasma. All cell lines were cultured at 37 °C with 5% CO_2_ in a humidified atmosphere unless differently stated.

### 4.2. Induced Pluripotent Stem Cells Induction and Validation

Earlier, established fully characterized control iPSC cell lines from anonymous donors (C1, C2 and C3) were used (Table S1) [53]. Fibroblasts from OI patients (P1, P2, and P3) were reprogrammed into iPSC lines as described previously [54]. Briefly, iPSCs were generated using the overexpression of *OCT4*, *SOX2*, *KLF4*, and *C-MYC* introduced by Sendai virus using the Sendai virus kit Invitrogen TM CytoTune TM- iPS 2.0 Sendai Reprogramming Kit (Life technologies, Carlsbad, CA, USA, A16518) [54]. iPSC lines were confirmed for pluripotency using PCR, immunocytochemistry (OCT3/4, SSEA4, TRA-1-60 and TRA-1-81), karyotyping, and/or trilineage differentiation (STEMdiff TM Trilineage differentiation, Stem cell, 05230). iPSCs were cultured with Essential 8 medium (E8, Gibco, Grand Island, NY, USA, A1517001) supplemented with 1% PEN/STREP (Gibco, Grand Island, NY, USA, 15140-122) on matrigel-coated six-well plates (Matrigel, six-well suspension culture plate, vWR, Radnor, PA, USA, 657185). Medium was refreshed daily, and cells were passaged once a week using Gentle Cell Dissociation reagent (StemCell Technologies, Vancouver, BC, Canada, 100-0485) according to the manufacturer’s protocol. Cells were split 1:10–1:50 to a new well for further maintenance. The iPSCs were cultured in an environment of 37 °C, 5% CO_2,_ and 5% O_2_ in a humidified atmosphere.

### 4.3. Induction of Induced Pluripotent Stem Cells into Induced Mesenchymal Stem Cells via Induced Neural Crest Cells’ Stage

The route of iPSCs to iNCCs differentiation is presented on Figure 1a,b. The iPSCs were harvested with accutase-solution (Millipore, Burlington, MA, USA, sf006) and seeded on Matrigel (hESC-qualified matrix, LDEV-free, Corning, New York, NY, USA, 354277) at 200,000–300,000 cells/well in a six-well plate (vWR, Radnor, PA, USA, 734-2323) containing Mouse embryonic fibroblast (MEF) conditioned media (CMMEF, R&D systems, Minneapolis, MN, USA, AR005) supplemented with PEN/STREP (Gibco, Grand Island, NY, USA, 15140-122). iNCC differentiation was initiated once the cells were at least 70% confluent with KSR medium. The KSR medium consist of knock-out medium, KSR (Gibco, Grand Island, NY, USA, 10828-028), MEM NEAA 100x (Gibco, Grand Island, NY, USA, 11140-035), GlutaMAX (Life technologies, Carlsbad, CA, USA, 35050-038), PEN/STREP (Gibco, Grand Island, NY, USA, 15140-122), and Beta-mercapto-ethanol (Gibco, Grand Island, NY, USA, 21985023) supplemented with 10 uM SB431542 (Bio-connect, Huissen, The Netherlands, S1067_50 mg), 1 uM Dorsomorphin (Selleckchem, Houston, TX, USA, S7306), and 1 uM CHIR99021 (Stemcell technologies, Vancouver, BC, Canada, 72052). Medium was changed on the first 3 days after which the KSR medium is slowly replaced by N2 medium on days 5, 7, 9, 11 (25%, 50%, 75%, 100%, respectively). N2 medium which consists of DMEM/F12 (Gibco, Grand Island, NY, USA, A1517001) supplemented with 1% N2 supplement (Life Technologies, Carlsbad, CA, USA, 17502001) and 1% PEN/STREP (Gibco, Grand Island, NY, USA, 15140-122). On day 12, the medium was replaced with N2 medium supplemented with 10 ng/mL FGF2 (PeproTech, Cranbury, NJ, USA, 100-18B) and 10 ng/mL EGF (PeproTech, Cranbury, NJ, USA, AF-100-15). The cells were harvested with accutase-solution (Millipore, Burlington, MA, USA, sf006) and seeded on 15 µg/mL Poly-L-ornithine hydrobromide (Sigma-Aldrich, Burlington, MA, USA, P3655-100 mg), 1µg/mL mLaminin (Sigma, Burlington, MA, USA, L2020-1 mg), and 10 µg/mL Fibronectin (Sigma, Burlington, MA, USA, F1141-2 mg) at 200,000 cells in a six-well plate (vWR, Radnor, PA, USA, 734-2323) for further maintenance. The iNCCs were cultured in an environment of 37 °C, 5% CO_2,_ and 5% O_2_ in a humidified atmosphere [20].

The iNCCs were plated on Matrigel at 200,000 cells in a six-well plate (vWR, Radnor, PA, USA, 734-2323) containing N2 medium. iMSC induction was started once the cells reach 50% confluency by replacing the medium with α-MEM+GlutaMAX (Gibco, Grand Island, NY, USA, 32561-029) supplemented with 10% FBS (Gibco, Grand Island, NY, USA, 10270-106), 1% PEN/STREP (Gibco, Grand Island, NY, USA, 15140-122), and 10 ng/mL FGF2 (PeproTech, Cranbury, NJ, USA, 100-18B). The medium was replaced twice a week for 21 days and split every week. The cells were cultured on Matrigel for the first two weeks and then seeded on plastic in a T75 flask (Nunc, Roskilde, Denmark, 156499). After differentiation, iMSCs were cultured in an environment of 37 °C and 5% CO_2_ in a humidified atmosphere.

### 4.4. Induction from Induced Mesenchymal Stem Cells to Osteoblast, Adipocyte, and Chondrocyte Cell Stage

The iMSCs were seeded in 12-well plate (Greiner bio-one, Frickenhausen, Germany, 665 180) with a density of 25,000 cells per well, after at least overnight cell attachment using the following conditions for 21 days. As the control medium, we used the culture iMSCs medium, consisting of α-MEM+GlutaMAX (Gibco, Grand Island, NY, USA, 32561-029) supplemented with 10% FBS (Gibco, Grand Island, NY, USA, 10270-106), 1% PEN/STREP (Gibco, Grand Island, NY, USA, 15140-122). As the osteogenic medium, we used α-MEM+GlutaMAX (Gibco, Grand Island, NY, USA, 32561-029), 90 µg/mL 2-phospho-L-ascorbic acid trisodium (Sigma, Burlington, MA, USA, 49752-10G), 5 mM B-glycerophosphate disodium salt hydrate (Sigma, Burlington, MA, USA, G5422-100G), 0.2% Heparin (LEO Pharma, Amsterdam, The Netherlands), 5% human platelet lysate (Stemcell thechnologies, 06962), 200 nM dexamethasone (Merck, Burlington, MA, USA, D4902-500 mg), and 1% PEN/STREP (Gibco, Grand Island, NY, USA, 15140-122). During differentiation, the culture medium was changed twice a week. After 21 days, the medium was removed and washed with DPBS (Gibco, Grand Island, NY, USA, 14190-094).

The adipogenic differentiation was performed as following: the iMSCs were seeded in a 24-well plate (Greiner bio-one, Frickenhausen, Germany, 662160) with a density of 20,000 cells per well. After 48 h, the cells were induced for 21 days. The adipogenic differentiation medium contained α-MEM+GlutaMAX (Gibco, Grand Island, NY, USA, 32561-029) supplemented with 10% FBS (Gibco, Grand Island, NY, USA, 10270-106), 3.5 mg/mL D-glucose (Merck, Burlington, MA, USA, 8337), 10 µg/mL insulin (Sigma, Burlington, MA, USA, I2643), 0.1 µM dexamethasone (Sigma, Burlington, MA, USA, D4902), 0.5 mM 3-Isobutyl-1-methylxanthine (IBMX, Sigma, Burlington, MA, USA, I5879), 200 µM indomethacin (Sigma, Burlington, MA, USA, I7378), and 1% PEN/STREP (Gibco, Grand Island, NY, USA, 15140-122). As non-induction medium during adipogenic and osteogenic induction experiments, the ordinary iMSC culture medium was used, consisting of α-MEM+GlutaMAX (Gibco, Grand Island, NY, USA, 32561-029) supplemented with 10% FBS (Gibco, Grand Island, NY, USA, 10270-106), 1% PEN/STREP (Gibco, Grand Island, NY, USA, 15140-122) for iMSCs, and MSC growth media 2 (PromoCell, Heidelberg, Germany, C-28009) for hMSCs. The culture medium was changed twice a week.

The chondrogenic differentiation was performed in accordance with the PromoCell protocol for the chondrogenic differentiation of primary MSCs. Briefly, the iMSCs were seeded in a 96-well U-bottom plate (Greiner bio-one, Frickenhausen, Germany, 650185) with a density of 200–300,000 cells per well. After 48 h, the chondrogenic induction was started with the chondrogenic differentiation medium (PromoCell, Heidelberg, Germany, C-28012) for three weeks. As the non-induction control medium, we used Dulbecco’s Modified Eagle’s medium–low glucose (Sigma-Aldrich, Burlington, MA, USA, D6046-500 mL) supplemented with 10% FBS (Gibco, Grand Island, NY, USA, 10270-106) and 1% PEN/STREP (Gibco, Grand Island, NY, USA, 15140-122) for iMSCs and MSC growth media 2 (PromoCell, Heidelberg, Germany, C-28009) for hMSCs. The culture medium was changed twice a week.

### 4.5. Staining of Osteogenic Differentiation

We performed two different stainings, Alkaline phosphatase and Alizarin red solution staining at day 21 and 28, as described elsewhere [55]. Briefly, after fixation with 10% formalin for 15 min at room temperature, the cells were washed twice with demi-water. For the ALP staining, the cells were incubated with NBT/BCIP (Sigma-Aldrich, Burlington, MA, USA, B6404-100 mL) until staining was satisfactory. Lastly, the cells were washed multiple times with demi-water and analyzed. For the AR staining, the cells were incubated with the 2% Alizarin red solution (Sigma-Aldrich, Burlington, MA, USA, A5533-25G) for at least 20 min at room temperature. Afterwards, the cells were washed with demi-water and analyzed. The amount of mineralization was qualitatively analyzed using microscopy.

### 4.6. Staining of Adipogenic Differentiation

After 21 days, the medium was removed and the cells were washed with DPBS (Gibco, Grand Island, NY, USA, 14190-094). The Sudan III staining stains red intracellular lipid vesicles, and it was performed in accordance with the PromoCell protocol. Shortly, cells were fixated for 30 min at room temperature with Saccomanno Fixation solution (Morphisto, Hessen, Germany, 13881.00250) and then washed with distilled water. Afterwards, the cells were incubated in 60% isopropanol for 5 min at room temperature. Subsequently, they were incubated with diluted Sudan III staining solution (Morphisto, Hessen, Germany, 10396.00500) at room temperature for 15 min. Finally, the cells were washed multiple times with distilled water and covered with PBS before being examined swiftly. Adipocyte morphology was scored using microscopy.

### 4.7. Staining of Chondrogenic Differentiation

After 21 days, the medium was removed and cells were washed twice with DPBS (Gibco, Grand Island, NY, USA, 14190-094). Alcian blue staining was performed in accordance with the PromoCell protocol. After fixation with the Saccomanno Fixation solution (Morphisto, Hessen, Germany, 13881.00250), for three hours at room temperature, the cells were washed twice with distilled water. The cell micromasses were stained in the dark for 45 min at room temperature with the Alcian blue staining solution (Sigma, Burlington, MA, USA, A3157). Next, the cells were washed twice with the destaining solution, consisting of 60% ethanol (98–100%) and 40% acetic acid (98–100%), for 10 min. Finally, micromasses were examined in the DPBS blue color indicative of proteoglycans. The staining was evaluated using microscopy.

### 4.8. Gene Expression Analysis

The total RNA was purified using a Quick-RNA miniprep kit of Zymo research (Zymo research, Irvine, CA, USA, R1055). The total RNA (140 ng) was transcribed to cDNA with a SuperScript Vilo cDNA synthesis kit (Invitrogen, Carlsbad, CA, USA, 11754050) in 20 µL final volume. The gene expression was tested using a quantitative real-time PCR (qPCR) analysis in 10 µL volume in duplicates with LightCycler^®^ 480 SYBR Green I Master (Roche, Basel, Switzerland, 4887352001) and performed with the LightCycler^®^ 480 System (Roche, Basel, Switzerland) according to the program presented in Table S5A. The list of primers (Integrated DNA technologies, IDT, Coralville, IA, US) is supplied in Table S5B. Raw data were analyzed with the LightCycler^®^ 480 Software, version 1.5 (Roche, Basel, Switzerland). Expressions of target genes were normalized towards the housekeeping TATA-box binding protein (*TBP*) gene. The relative gene expression was calculated with the ΔΔCt method.

### 4.9. Flow Cytometry and Analysis

Approximately 300,000 iNCCs were centrifuged at 300× *g* for 5 min. The supernatant was removed, and the samples were stained for 30 min at 4 °C with a viability staining (Table S6). After washing with PBS, cells were incubated with 1% heat-inactivated (HI) FBS (Gibco, Grand Island, NY, USA, 10270-106) for again 30 min at 4 °C. After blocking, the cells were stained with primary surface antigen antibodies, P75 and CD57 (Table S6), for 30 min at 4 °C in the dark. Afterwards, the cells were washed twice with staining buffer (DPBS (Gibco, Grand Island, NY, USA, 14190-094) + 1% HI-FBS). The cells were fixed and permeabilized by incubating with the fixation/permeabilization solution (BD biosciences, Franklin Lakes, NJ, USA, 554714) for 20 min at 4 °C. Subsequently, the cells were washed twice with BD Perm/Wash buffer (BD biosciences, Franklin Lakes, NJ, USA, 554714). Then, the cells were incubated with the primary antibodies for the intracellular protein PAX6 (Table S6) for 30 min at 4 °C in the dark. Lastly, the cells were washed twice more with BD Perm/Wash buffer (BD biosciences, Franklin Lakes, NJ, USA, 554714), and, finally, they were resuspended in staining buffer prior to flow cytometry analysis. The flow cytometry acquisition was performed on a Attune CytPix Flow Cytometer (Lasers: BRVY).

The analysis was performed using FlowJo (v10.7.1, Ashland, OR, US) on Windows 10. All fcs files were automatically compensated using the corresponding single-color controls. Fluorescence minus one (FMO) controls were used to determine the positive gate of each marker. The percentage of P75NTR, HNK-1, and PAX6 positive population in stained iNCCs was further analyzed using the percentage of the parent gate. The gating strategy can be found below and was consistently used for all flow cytometry data, including the control and patients’ fcs files. The cleaning gates used prior to define the population of interest include FSC-H vs. FSC-A; to remove the doublets, FSC-A/SSC-A; Live dead marker, to discriminate the viable iNCCs; SSC-A vs. FSC-A, used to remove debris and select the population of interest.

### 4.10. Statistical Analysis

mRNA expression data were analyzed using two-way ANOVA and one-way ANOVA GraphPad Prism version 9 (GraphPad Software Inc., San Diego, CA, USA). Graphs were generated using GraphPad Prism version 9 (GraphPad Software Inc., San Diego, CA, USA).

## 5. Conclusions

In conclusion, we generated iMSCs and subsequent osteoblast-like cells from both control and OI patient cell lines. The iMSCs presented the potential to generate osteoblast-like cells, early adipocytes, and chondrocytes. Although we noted differences between control and patient cell lines in osteogenic and adipogenic potential, such as increased adipogenic potential in patient iMSCs, reduced chondrogenic gene expression in DN OI iMSCs, and osteoblast properties attributed to OI pathology (reduced collagen expression and mineralization), further characterization is still needed to explore the differences between the control, HI, and DN OI patient cell lines. Creation of patient-specific iMSCs provides a minimally invasive model, overcoming the limitations of primary cell culture OI models and opening new possibilities to examine patient-specific osteoblast-like cells, and their collagen production and processing, screen pharmaceutical compounds, and develop therapies in a patient-specific molecular setting.

## Figures and Tables

**Table 1:** The percentages of single-stain (P75NTR+, HNK-1+ and PAX6+) and double-stained cells (P75NTR+/HNK-1+ and P75NTR+/PAX6+) in healthy control (C1, C2, C3) and OI patients (P1, P2, and P3) induced neural crest cells (iNCCs) from flow cytometry (FACS) analysis.

**Cell Line**	**P75NTR+**	**P75NTR+/HNK-1+**	**HNK-1+**	**P75NTR-/HNK-1−**
C1	0.70%	0.86%	72.5%	26.0%
C2	2.57%	2.97%	73.4%	21.1%
C3	52.0%	1.76%	4.48%	41.8%
P1	67.8%	3.47%	0.84%	27.9%
P2	24.4%	1.13%	2.37%	72.1%
P3	46.6%	2.18%	9.68%	41.6%
**Cell Line**	**P75NTR+**	**P75NTR+/PAX6+**	**PAX6+**	**P75NTR-/PAX6−**
C1	1.38%	0.18%	7.25%	91.2%
C2	3.38%	2.16%	13.1%	81.4%
C3	52.7%	1.00%	0.52%	45.7%
P1	69.7%	1.49%	0.22%	28.5%
P2	23.9%	1.58%	1.06%	73.4%
P3	48.1%	0.69%	0.39%	50.9%

## Data Availability

All relevant data can be found within the article and its supplementary information. Further inquiries can be directed to the corresponding author.

## Supplementary Materials

The following supporting information can be downloaded at https://www.mdpi.com/article/10.3390/ijms25063417/s1.
