# Supplementary material for: In Vitro Modelling of Osteogenesis Imperfecta with Patient-Derived Induced Mesenchymal Stem Cells
Source: Int J Mol Sci. 2024 Mar 18;25(6):3417. doi: 10.3390/ijms25063417 (PMC10970641; doi:10.3390/ijms25063417)
Supplement: Supplementary file 1 [file ijms-25-03417-s001.zip › ijms-2897673-supplementary.pdf]

Supplemental Figures and Tables

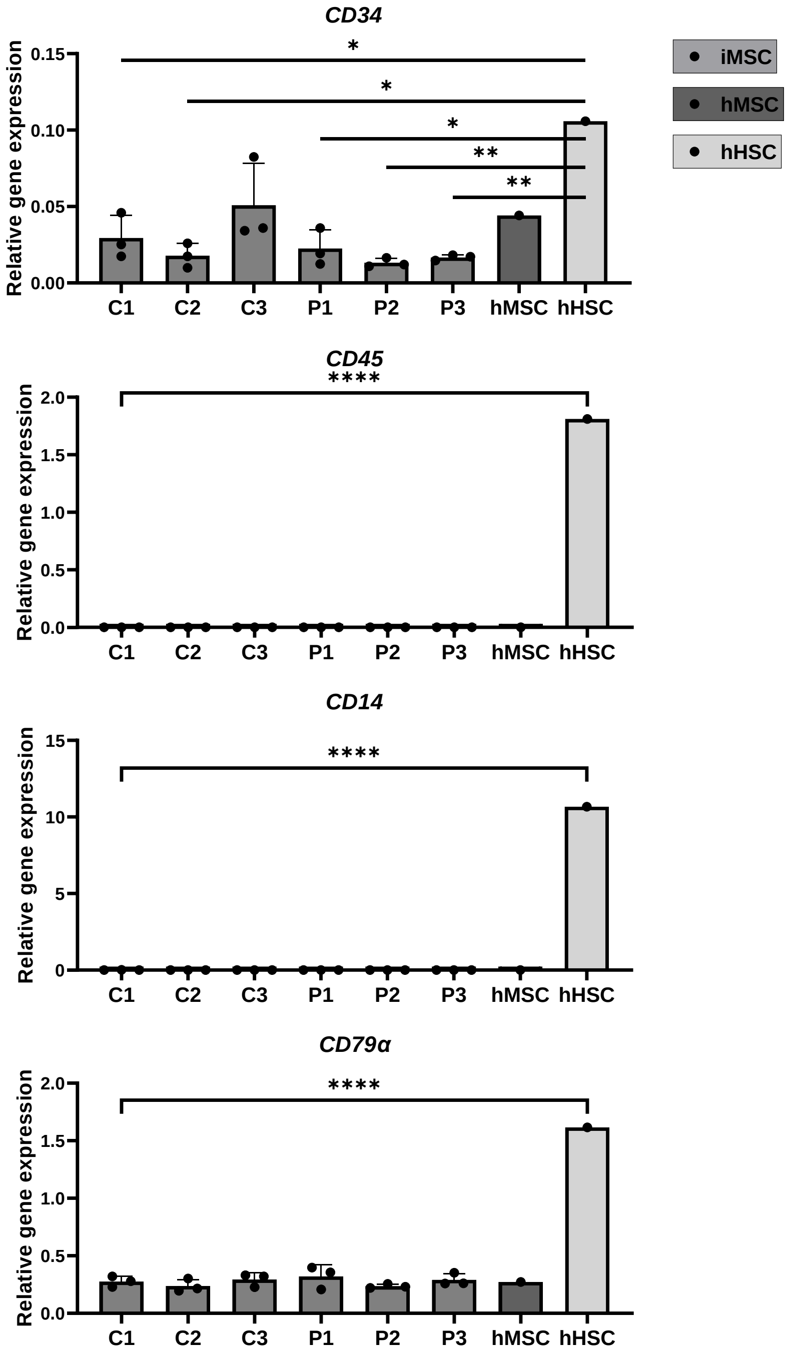

Figure S1

Relative gene expression of hematopoietic stem cell (hHSC) markers *CD45, CD34, CD14* and *CD79α* in healthy controls (C1, C2, C3), OI patients (P1, P2 and P3), primary bone marrow mesenchymal stem cells (hMSC) and primary hematopoietic stem cells (hHSC). Relative gene expression was normalized against *TBP*. Standard deviation (SD) is shown per cell line (n=3), no SD is present for hHSC and hMSC (n=1). Statistical significance is indicated on the graphs (P-values ≤0.05 (*), ≤ 0.01 (**), ≤0.0001 (****)).

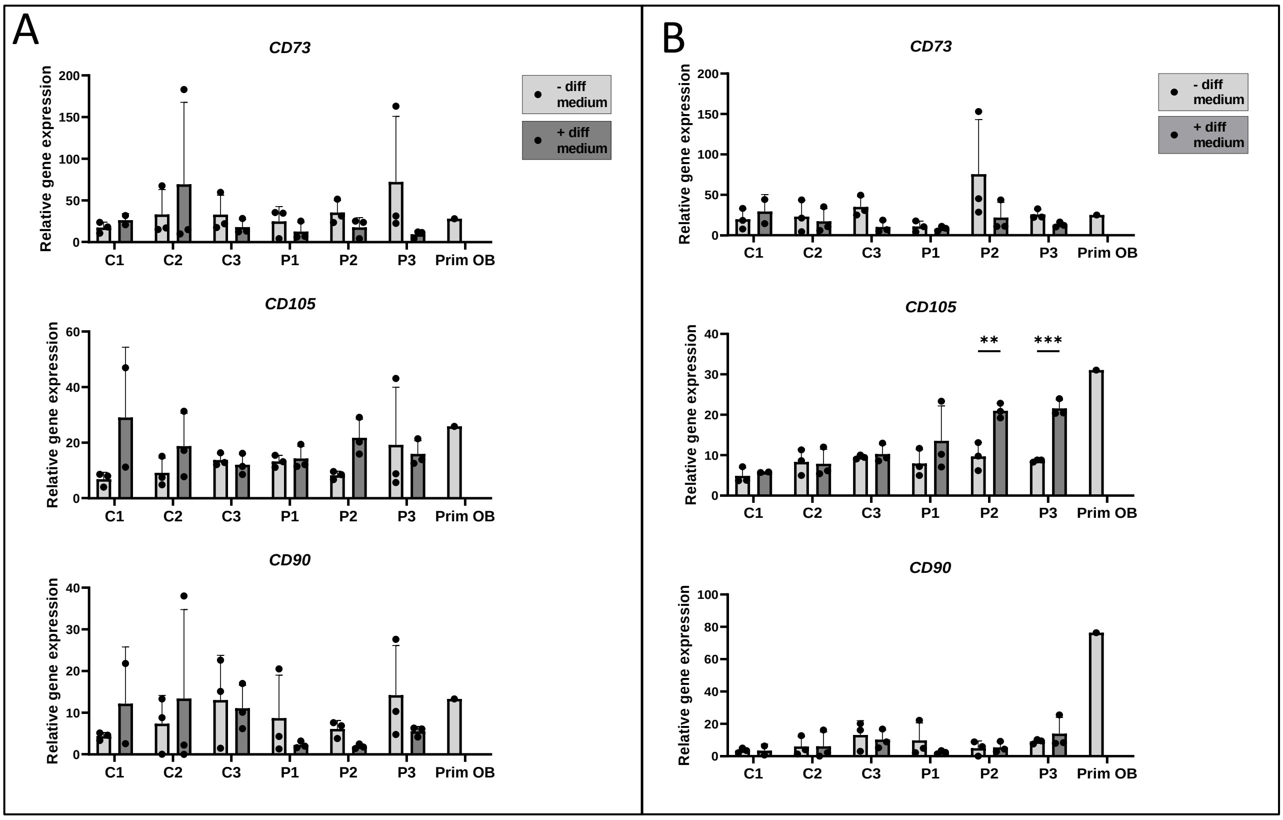

Figure S2

Relative gene expression of mesenchymal stem cell markers *CD73*, *CD90* and *CD105* in osteogenic induced and not induced healthy control iMSC (C1, C2, C3), OI patient iMSCs (P1, P2 and P3) and not induced primary osteoblasts (Prim OB) on day 21 (A) and day 28 (B). Relative gene expression was normalized against *TBP*. Standard deviation (SD) is shown per cell line (n=3), no SD is present for Prim OB (n=1). Statistical significance is indicated on the graphs (P-values ≤ 0.01 (**), ≤ 0.001 (***)).

Table S1

Information about healthy control iPSC (C1, C2, C3) and OI patient iPSC (P1, P2 and P3) cell lines.

Line	Name	Genotype	Pathogenic variant	Sex	Age	Reprogramming method	Reference	Characterizations
C1	Hvs228	Control	NA	F	19 y	Lentivirus	Nadadhur, A. G. *et al* [46]	Karyotyping, PCR
C2	Hvs420A	Control	NA	M	21 y	Lentivirus	VUi036-A	Staining of OCT3/4, SSEA4, AP, TRA-1-60 and TRA-1-81
C3	Hvs451A	Control	NA	M	19 y	Sendai	VUi032-A	Staining of OCT3/4, SSEA4, TRA-1-60 and TRA-1-81, PCR Sendai and karyotyping
P1	Hvs492A	HI OI type I	*COL1A1*: c.279delT	M	38 y	Sendai	VUi043-A	Staining of OCT3/4, SSEA4, TRA-1-60 and TRA-1-81, and 3 germ layer test (PCR)
P2	Hvs459	DN OI type 3	*COL1A1*: c.2299G>A	F	0y	Sendai	VUi045-A	Staining of OCT3/4, SSEA4, AP, TRA-1-60 and TRA-1-81, PCR Sendai and 3 germ layer test (staining)
P3	Hvs460	DN OI type 4	*COL1A1*: c.1678G>A	F	0y	Sendai	VUi046-A	Staining of OCT3/4, SSEA4, TRA-1-60 and TRA-1-81, PCR Sendai and 3 germ layer test (staining)

Table S2

Qualitative analysis of alkaline phosphatase (ALP) and Alizarid Red (AR) staining on day 21 and day 28 after osteogenic induction in healthy controls iMSC (C1, C2, C3), OI patients iMSCs (P1, P2 and P3) and hMSCs. ALP and AR stainings were rated by + (some cells are stained), ++ (most/all cells are stained), – (no cells are stained) and x (no cells present).

**Osteoblast differentiation**				
**Cell line**	ALP D21	ALP D28	AR D21	AR D28
**C1**	+	+	+	+
**C1**	++	++	+	+
**C1**	++	++	+	++
**C2**	++	++	+	+
**C2**	+	+	+	++
**C2**	+	+	-	-
**C3**	++	++	-	-
**C3**	++	++	-	++
**C3**	++	x	-	x
**P1**	++	-	-	-
**P1**	++	++	-	-
**P1**	++	++	+	+
**P2**	+	+	+	+
**P2**	++	x	+	x
**P2**	x	++	+	x
**P3**	+	++	-	+
**P3**	++	x	+	x
**P3**	+	++	+	-
**hMSC**	++	++	++	++

Table S3

Qualitative analysis of Sudan III staining after adipogenic induction in healthy control iMSC (C1, C2, C3), OI patient iMSCs (P1, P2 and P3) and hMSCs. Morphology is rated by + (large cells with normal nucleus), ++ (large cells with big nucleus) and – (spindle-like cells), while the presence of vesicles is rated by + (some vesicles are present, small), ++ (a lot of vesicles are present, small), +++ (many vesicles are present, big) and – (no vesicles are present).

**Adipogenic differentiation**		
**Cell line**	**Morphology**	**Vesicles**
**C1**	++	+
**C1**	++	+
**C1**	++	+
**C2**	++	-
**C2**	++	++
**C2**	+	-
**C3**	++	++
**C3**	++	++
**C3**	++	++
**P1**	++	++
**P1**	++	++
**P1**	++	++
**P2**	++	++
**P2**	++	++
**P2**	++	+++
**P3**	++	-
**P3**	++	-
**P3**	++	++
**hMSC**	++	+++

Table S4

Qualitative analysis of Alcian blue staining in healthy controls iMSC (C1, C2, C3) and OI patient iMSCs (P1, P2 and P3) after chondrogenic induction. Staining was rated by + (lightly blue), ++ (blue) and – (no staining). The presence of cell monolayer outgrowth was also noted by + (present) and – (absent).

	Alcian blue staining	Cell monolayer outgrowth
**C1**	+	-
**C1**	++	-
**C1**	++	-
**C2**	+++	-
**C2**	++	-
**C2**	+	-
**C3**	++	-
**C3**	++	-
**C3**	++	-
**P1**	+	-
**P1**	++	-
**P1**	++	-
**P2**	++	-
**P2**	++	-
**P2**	+	-
**P3**	+	-
**P3**	++	-
**P3**	++	-

Table S5

(a) RT-qPCR analysis qPCR programme

	Tm (°C)	Time(s)	Ramp rate (°C/s)	Cycli	Analysis Mode
Preheat	95	10 min	4.8	1 ×	None
PCR	95<br>60<br>72<br>78	10<br>5<br>10<br>5	4.8<br>2.5<br>4.8<br>4.8	45x	Quantification
Melting-curve	95<br>60<br>95	1<br>1	4.8<br>2,2<br>0,03	1x	Melting curves
Cooling	40	30	1,5	1×	None

(B) List of primers used in RT-qPCR analysis to measure the relative mRNA expression levels.

**Gene**	**Forward**	**Reverse**	**Product length (bp)**
ACAN	GCACCTTTCAGTGTCCATTCC	GTGGTCATAGTTCACCTTGAAGA	258
ALP	AGGGACATTGACGTGATCAT	CCTGGCTCGAAGAGACC	242
CD105	CACCACAGCGGAAAAAGGTG	GCCGGTTTTGGGTATGGGTA	335
CD73	CTCCTCTCAATCATGCCGCT	CCCAGGTAATTGTGCCATTGT	182
CD90	TCCCGAACCAACTTCACCAG	ATGCCCTCACACTTGACCAG	173
COL10A1	GGGCAGAGGAAGCTTCAGAAA	TCTCAGATGGATTCTGCGTGC	73
COL1A1	GTGCTAAAGGTGCCAATGGT	ACCAGGTTCACCGCTGTTAC	128
COL2A1	AGGATGGCTGCACGAAACAT	GCCATTCAGTGCAGAGTCCT	255
FABP4	TGACGAAGTCACTGCAGATG	GAACTTCAGTCCAGGTCAAC	217

HNK-1/B3GAT1	TGGGTTGTGAGTGCTGGTAA	GTGATGAGCAGAGTCCAGGG	116
NANOG	AATGGTGTGACGCAGAAGGC	TGCACCAGGTCTGAGTGTTC	196
NGFR/p75NTR	GATCCCTGGCCGTTGGATTA	TTGCAGCTGTTCCACCTCTTG	267
OCN/BGLAP	CACCGAGACACCATGAGAGC	CTGCTTGGACACAAAGGCTGC	132
OCT4	CCCACACTGCAGCAGATCA	ACCACACTCGGACCACATCC	70
OPN	TTCCAAGTAAGTCCAACGAAAG	GTGACCAGTTCATCAGATTCAT	181
PAX6	AGGTCAGGCTTCGCTAATGG	TGCTGATTGGTGATGGCTCA	97
PPARγ	CGACCAGCTGAATCCAGAGT	GATGCGGATGGCCACCTCTT	241
RUNX2	CGTGGCCTTCAAGGTGGTAG	GAGGCATTCCGGAGCTCAG	105
SOX2	ATGGGTTCGGTGGTCAAGTC	CTGATCATGTCCCGGAGGTC	107
SOX9	GGCAAGCTCTGGAGACTTCTG	CCCGTTCTTCACCGACTTCC	138
TBP	AGTTCTGGGATTGTACCGCA	TCCTCATGATTACCGCAGCA	139
TFAP2A	GACCTCTCGATCCACTCCTTAC	GAGACGGCATTGCTGTTGGACT	137

Table S6

List of antibodies and stainings used during flow cytometry analysis of healthy control iNCC (C1, C2, C3) and OI patient iNCC (P1, P2 and P3) cell lines.

**Name**	**Fluorophore**	**Company**	**Catalogue number**	**Concentration**	**Clone**
Anti-p75 NGF Receptor antibody [NGFR5]	PE-Cy7	Abcam	ab234270	1:100	NGFR5
CD57 Antibody	FITC	Miltenyi Biotec	130-122-935	1:100	TB03
PAX-6 Antibody, REAfinity™	APC	Miltenyi Biotec	130-123-328	1:100	REA507

Fixable Viability Dye	eFluor780	eBioscience, ThermoFisher Scientific	65-0865-14	1:1000	-

APC allophycocyanin, FITC fluorescein isothiocyanate, PE R-phycoerthrin.
